# Cervical Intraepithelial Neoplasia grade 2 biopsy: Do p16INK4a and Ki-67 biomarkers contribute to the decision to treat? A cross-sectional study

**DOI:** 10.1590/1516-3180.2022.0527.R2.280423

**Published:** 2023-08-25

**Authors:** Amanda Leal Ferreira, Nasle Domingues Dibe, Bruna Rodrigues de Paiva, Elyzabeth Avvad Portari, Dione Corrêa de Araújo Dock, Nilma Valéria Caldeira Ferreira, Saint Clair Gomes, Fábio Bastos Russomano, Cecília Vianna de Andrade

**Affiliations:** I MSc. Biomedical and PhD Student, Laboratory of Diagnosis Pathology and Cytopathology, Instituto Nacional de Saúde da Mulher, da Criança e do Adolescente Fernandes Figueira (IFF), Fundação Oswaldo Cruz (FIOCRUZ), Rio de Janeiro (RJ), Brazil.; II MD. Gynecologist, Laboratory of Diagnosis Pathology and Cytopathology, Instituto Nacional de Saúde da Mulher, da Criança e do Adolescente Fernandes Figueira (IFF), Fundação Oswaldo Cruz (FIOCRUZ), Rio de Janeiro (RJ), Brazil.; III MD. Postgraduate Student in Nutrology, Laboratory of Diagnosis Pathology and Cytopathology, Instituto Nacional de Saúde da Mulher, da Criança e do Adolescente Fernandes Figueira (IFF), Fundação Oswaldo Cruz (FIOCRUZ), Rio de Janeiro (RJ), Brazil.; IV MD, MSc, PhD. Pathologist, Researcher in Laboratory of Diagnosis Pathology and Cytopathology, Instituto Nacional de Saúde da Mulher, da Criança e do Adolescente Fernandes Figueira (IFF), Fundação Oswaldo Cruz (FIOCRUZ), Rio de Janeiro (RJ), Brazil.; V MD. Physician and Pathologist, Laboratory of Diagnosis Pathology and Cytopathology, Instituto Nacional de Saúde da Mulher, da Criança e do Adolescente Fernandes Figueira (IFF), Fundação Oswaldo Cruz (FIOCRUZ), Rio de Janeiro (RJ), Brazil.; VI BSc. Immunohistochemistry Technician, Laboratory of Diagnosis Pathology and Cytopathology, Instituto Nacional de Saúde da Mulher, da Criança e do Adolescente Fernandes Figueira (IFF), Fundação Oswaldo Cruz (FIOCRUZ), Rio de Janeiro (RJ), Brazil.; VII BSc, PhD. Researcher in Clinical Research Unit, Instituto Nacional de Saúde da Mulher, da Criança e do Adolescente Fernandes Figueira (IFF), Fundação Oswaldo Cruz (FIOCRUZ), Rio de Janeiro (RJ), Brazil.; VIII MD, MSc, PhD. Gynecologist, Center for the Clinical and Surgical Care of Women, Instituto Nacional de Saúde da Mulher, da Criança e do Adolescente Fernandes Figueira (IFF), Fundação Oswaldo Cruz (FIOCRUZ), Rio de Janeiro (RJ), Brazil.; IX MD, MSc, PhD. Pathologist, Laboratory of Diagnosis Pathology and Cytopathology, Instituto Nacional de Saúde da Mulher, da Criança e do Adolescente Fernandes Figueira (IFF), Fundação Oswaldo Cruz (FIOCRUZ), Rio de Janeiro (RJ), Brazil.

**Keywords:** Uterine cervical dysplasia, Cyclin-dependent kinase inhibitor p16, Ki-67 antigen, Cervical intraepithelial neoplasia, Cervical intraepithelial neoplasia grade 2, CIN2, p16INK4a, Ki-67

## Abstract

**BACKGROUND:**

Managing cervical intraepithelial neoplasia grade 2 (CIN2) is challenging, considering the CIN2 regression rate, perinatal risks associated with excisional procedures, and insufficient well-established risk factors to predict progression.

**OBJECTIVES:**

To determine the ability of p16INK4a and Ki-67 staining in biopsies diagnosed with CIN2 to identify patients with higher-grade lesions (CIN3 or carcinoma).

**DESIGN AND SETTING:**

Cross-sectional study conducted at a referral center for treating uterine cervical lesions.

**METHODS:**

In 79 women, we analyzed the correlation of p16INK4a and Ki-67 expression in CIN2 biopsies with the presence of a higher-grade lesions, as determined via histopathology in surgical specimens from treated women or via two colposcopies and two cytological tests during follow-up for untreated women with at least a 6-month interval. The expression of these two biomarkers was verified by at least two independent pathologists and quantified using digital algorithms.

**RESULTS:**

Thirteen (16.8%) women with CIN2 biopsy exhibited higher-grade lesions on the surgical excision specimen or during follow-up. p16INK4a expression positively and negatively predicted the presence of higher-grade lesions in 17.19% and 86.67% patients, respectively. Ki-67 expression positively and negatively predicted the presence of higher-grade lesions in 40% and 88.24% patients, respectively.

**CONCLUSIONS:**

Negative p16INK4a and Ki67 immunohistochemical staining can assure absence of a higher-grade lesion in more than 85% of patients with CIN2 biopsies and can be used to prevent overtreatment of these patients. Positive IHC staining for p16INK4a and Ki-67 did not predict CIN3 in patients with CIN2 biopsies.

## INTRODUCTION

The incidence and mortality rates of uterine cervical carcinoma have decreased considerably over the years in regions where systematic screening programs have been implemented.^1^ Colposcopy assessment is necessary depending on the cytology results, and in some cases the decision to treat the patient requires biopsy proof of a precursor lesion. Treatment is indicated when a biopsy specimen is diagnosed as cervical intraepithelial neoplasia (CIN) grade 3 or higher, and cases with CIN1 should be monitored.^2^

The decision to treat women with biopsy showing CIN grade 2 is currently under debate,^3,4,5,6,7,8^ considering the regression rate in this grade^7^ and the perinatal risks associated with excisional procedures.^9,10^ The Brazilian Guidelines for Cervical Cancer Screening recommend that CIN2 biopsy in women younger than 25 years can be managed conservatively,^3,4^ whereas surgical excision of such lesions is recommended in older women.^2^ However, consistent data suggest that it is safe and reasonable to manage CIN2 conservatively in women under 30 years due to the benign prognosis.^5,7^ However, the regression rate of CIN2 support conservative management in selected patients, regardless of age, considering other factors, such as severity of referral cytology and human papillomavirus (HPV) status.^8,11^

Although the current discussion on the decision to treat CIN2 is based on prognosis, another relevant question is the limited ability of a directed biopsy to represent the entire lesion.^12,13^ The patient may be treated inadequately if the biopsy is not targeted to the more severe colposcopic alterations. This is an advantage of excisional treatment over destructive treatment: the entire transformation zone can be accessed, and the final diagnosis can be provided, thereby ruling out an unsuspected microinvasion. Another limitation of CIN2 biopsies is the moderate level of inter-observer agreement in this diagnosis, which leads to uncertainty if the result is actually CIN2.^14,15^

Therefore, the decision to avoid treating patients with CIN2 biopsies should be based on the low probability of a higher-grade lesion underdiagnosed by directed biopsy, rather than avoiding its progression to carcinoma.

Given the limitations of histopathological diagnosis for CIN, a set of recommendations called the lower anogenital squamous terminology (LAST) was formulated by the American College of Pathologists and the American Society of Colposcopy and Cervical Pathology in 2012. These guidelines recommend the assessment of the staining pattern of p16INK4a to classify this diagnostic category as either high- or low-grade lesions, and recommend treatment in CIN2 p16INK4a-positive patients and follow-up in CIN2 p16INK4a negative patients.^16^ However, the sole use of this biomarker is insufficient for the decision to treat patients with CIN2 biopsy. Using p16INK4a can improve the level of inter-observer agreement in the diagnosis of CIN2.^17^

Ki-67, another biomarker related to CIN grade,^18^ is used in association with p16INK4a in dual-staining cytology to detect HSIL in cytology specimens.^19^ Ki-67 has also been used to identify patients with CIN2 lesions that are more likely to progress to carcinoma^20,21^ and can predict CIN grade 3 or higher in women with a CIN2 biopsy.

Considering the limitations of directed biopsies and the need to identify women where a CIN2 biopsy could underrepresent more severe lesions, the current study assessed the reliability of p16INK4a and Ki-67 in predicting the presence of higher-grade lesions and their potential usefulness in deciding whether to treat these patients.

## OBJECTIVE

This study aimed to determine the ability of p16INK4a and Ki-67 staining in CIN2 directed biopsies to identify women with higher-grade lesions (CIN3 or carcinoma).

## METHODS

### Study design

This was a cross-sectional study in a retrospective cohort.

### Eligibility criteria

CIN2 cervical lesions were identified in laboratory files at a referral center for uterine cervical pathology, where patients with altered cytology were evaluated via colposcopy, as recommended in the Brazilian Guidelines, from January 2006 to December 2016.

### Inclusion criteria

Women with a CIN2 diagnosis confirmed using cervical biopsy, availability of paraffin-embedded samples for immunohistochemical analyses, and diagnostic data on surgical specimens for treated women or follow-up data (with two colposcopies and two cytologies with a minimum six-month interval) for untreated women.

### Exclusion criteria

In cases where the biopsies were excised, the original lesion was undetectable on immunohistochemistry (IHC) slides, and the diagnosis of CIN2 was not confirmed by the review pathologists. Biopsies where the entire lesion was removed according to the colposcopist’s judgment were considered excisional. Untreated women who did not attend six-monthly visits for at least one year were considered lost to follow-up.

### Clinical protocol

Biopsies were performed in patients referred for colposcopy based on cytological atypia obtained from the primary care unit (referral cytology data). Biopsies were performed in patients in whom colposcopy diverged from primary care cytology findings: major colposcopy findings in women with cytology suggestive of low-grade lesion, atypical squamous cells of undetermined significance (ASC-US), minor abnormal findings in women with cytology suggestive of high-grade lesions, or atypical squamous cells-cannot exclude high-grade squamous intraepithelial lesion (ASC-H). Patients with findings consistent with high-grade lesions, cytology findings suggestive of high-grade lesions or ASC-H, and transformation zone type I or II were treated by excision without biopsy, and their specimens were not eligible for inclusion.

Patients with CIN2 biopsy results underwent excisional treatment unless they had transformation zone type I or II and were younger than 25 years, or in cases in which abnormal findings that persisted after biopsy suggested the absence of disease or less severe lesions.

Untreated patients were followed-up with six-monthly cytology and colposcopy for 12 months. During follow-up, a new biopsy or treatment was performed if new cytology or colposcopy findings suggested persistence or progression of the lesion.

### Immunohistochemical analysis

Ki-67 immunohistochemical (IHC) expression was assessed using a monoclonal antibody (1:200 dilution; clone SP6, Cell Marque, Rocklin, United States) on the Max Polymer Detection System (BOND-MAX, Leica, Melbourne, Australia). p16INK4a expression was analyzed using an anti-p16INK4a prediluted mouse monoclonal antibody (1:30 dilution; CINtec p16INK4a Histology, Ventana Medical Systems, Tucson, United States) on an automated IHC staining system.

For the positive expression control, tissue microarray slides were prepared in samples positive for p16INK4a and Ki-67, according to Pires et al.^22^ For the negative control, the primary antibody was replaced with phosphate-buffered saline.

### Review of histology slides and evaluation of IHC staining

To improve the reliability of CIN2 diagnosis, histological data and p16INK4a and Ki-67 IHC expression were analyzed independently by three experienced pathologists who were blinded to the clinical data. The slides were evaluated separately in the following order: hematoxylin and eosin-, p16INK4a-, and Ki-67-stained slides.

The samples were classified histologically as non-CIN (without lesions), CIN1, CIN2, and CIN3. IHC staining of p16INK4a was evaluated by three independent pathologists according to LAST recommendations.^16^ Strong staining with continuous distribution in at least one-third of the epithelium was considered positive and focal or multifocal (irregular), weak, or absent staining was considered negative. The review and IHC staining diagnosis for each case was defined by the consensus of at least two evaluators. Cases of doubtful diagnosis were jointly reviewed by three pathologists.

In assessing Ki-67 IHC staining, pathologists were instructed to define Ki-67 as positive when more than 50% of cells were stained. Although consensus on the cutoff value for positive Ki-67 expression is not available, 50% was chosen because it is associated with presence of CIN3 lesions or carcinoma.^20,21^

Unlike p16INK4a analysis, which is a qualitative assessment, Ki-67 analysis is based on the percentage of stained nuclei and digital quantification is used to define Ki-67 status. The slides were scanned using an Aperio ScanScope CS5 scanner (Leica, Vista, United States). Ki-67 expression was quantified using the Aperio ImageScope software version 11 (Leica, Vista, United States) and IHC Nuclear algorithm version 1 (Leica, Vista, United States) within the area of quantification demarcated by one of the participating pathologists.

### Definition of the presence of a higher-grade lesion

Higher-grade lesions were determined in the surgical specimens of treated patients. CIN3 or carcinoma detected during the first year after biopsy was considered a diagnosis missed by directed biopsy, rather than progression. Untreated women were followed-up with cytology and colposcopy analyses every six months. During this period, areas of suspected higher-grade lesions were either biopsied or the patients underwent an excisional procedure. Women with negative or minor alterations in cytology or colposcopy and not biopsied were considered as not having a higher-grade lesion. The results were classified as ≥ CIN3 (CIN3 lesions and carcinomas) or ≤ CIN2 (healthy tissue or CIN1 and CIN2 lesions).

### Data retrieval, management, and statistical analysis

Clinical and follow-up data were obtained from medical records and cytology and histological reports. Data were stored in electronic spreadsheets (Microsoft Excel, version 2013) and processed using the OpenEpi software version 3.01 (https://www.openepi.com/Menu/OE_Menu.htm). Potential associations between clinical features and biomarkers and presence of CIN3 or carcinoma were tested using odds ratios (ORs) and the respective 95% confidence intervals (CIs). Data were evaluated by excluding missing data. Significance of associations was defined as a p-value less than 0.05 (Table 1).

**Table 1.** Characteristics of included patients in relation to referral cytology, colposcopy, immunohistochemistry biomarkers, and final diagnosis

Clinical features and biomarker status	Final diagnosis		Total n (%)	OR	P
	≤ **CIN2 (%)**	≥ **CIN3 (%)**			
**Age (mean ± SD)**	32.47 ± 10.17	40.00 ± 14.82	33.71 ± 11.31		
< 25 years	14 (82.3)	3 (17.7)	17 (21.5)	ref	
≥ 25 years	52 (83.9)	10 (16.1)	62 (78.5)	0.9 (0.2–4.54)	1.0^a^
< 30 years	32 (86.5)	5 (13.5)	37 (46.8)	Ref	
≥ 30 years	34 (80.9)	8 (19.1)	42 (53.2)	1.5 (0.4–5.51)	0.51^b^
**Referral cytology**					
Negative	4 (80.0)	1 (20.0)	5 (6.3)		
ASC-US	5 (71.4)	2 (28.6)	7 (8.9)		
LSIL	14 (70.0)	6 (30.0)	20 (25.3)	ref^d^	
ASC-H^c^	12 (100.0)	0	12 (15.2)		
HSIL	31 (88.6)	4 (11.4)	35 (44.3)	0.3 (0.06–0.86)	0.02^c^
**Colposcopy**					
Minor alterations	40 (83.3)	8 (16.7)	48 (60.8)	ref	
Major alterations	25 (83.3)	5 (16.7)	30 (38.0)		
Suspected invasion	1 (100.0)	0	1 (1.3)	0.97 (0.23–3.31)	1.0^b^
**Transformation zone**					
Type 1 or 2	52 (85.2)	9 (14.8)	61 (77.2)	ref	
Type 3	13 (76.5)	4 (23.5)	17 (21.5)	1.64 (0.38–6.1)	
Not available^e^	1 (100.0)	0	1 (1.3)		0.39^c^
**p16**					
Negative	13 (86.7)	2 (13.3)	15 (19.0)	ref	
Positive	53 (82.8)	11 (17.2)	64 (81.0)	1.35 (0.29–9.9)	1.0^b^
**Ki-67**					
Negative	60 (88.2)	8 (11.8)	68 (86.1)	ref	
Positive	6 (60.0)	4 (40.0)	10 (12.7)	4.85 (1.0–21.97)	0.04^b^
Not available^e^	0	1 (100.0)	1 (1.3)		
**Total**	**66 (83.5)**	**13 (16.5)**	**79 (100.0)**		

CIN = cervical intraepithelial neoplasia; OR = odds ratio; SD = standard deviation; ^a^Fisher´s Exact test; ^b^chi-square; ASCUS-US = atypical cells of undetermined significance; LSIL = low-grade intraepithelial lesion; ASC-H = atypical cells of undetermined significance, ^c^cannot rule out high-grade intraepithelial lesion; HSIL = high-grade intraepithelial lesion; ref = reference, ^d^negative + ASC-US + LSIL; ^e^Total losses in the variable.

### Ethical compliance

The research protocol was approved on May 8, 2018, by the Institutional Review Board (IRB) of the Oswaldo Cruz Foundation (protocol no. 2.533.682 IFF-FIOCRUZ). As this was a retrospective study using paraffin-embedded tissue samples and clinical data obtained from clinical files in previous research with no risk to patients, informed consent was waived by the IRB.

### Statement of patient and public involvement

There was no patient and public involvement in the study.

## RESULTS

**Figure 1.**
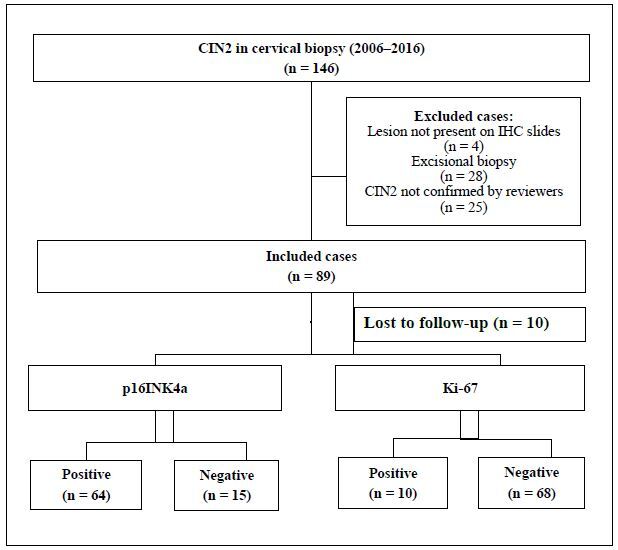
Flowchart of sample selection and biomarker status.

We identified 146 cases of CIN2 lesions in the laboratory database from 2006 to 2016. Here, 89 patients were included in the analysis, and 10 untreated women were excluded because they had not completed the one-year follow-up period (Figure 1). Mean age of the included women was 33.7 years (range, 17.8–79.0 years) and median age was 31.4 years (Table 1). No significant differences were observed in age, referral cytology, or colposcopy findings among included patients and those who were excluded or lost to follow-up (data not shown).

Twenty-three patients (29.1%) with median age 26.9 years did not undergo treatment, whereas 56 patients (70.9%) with median age 34.9 years underwent treatment. In the latter group, the time between biopsy and treatment was 112 days (Table 1).

None of the untreated patients presented with CIN3 or carcinoma after one-year follow-up. CIN3 was diagnosed in 13 cases (16.5% of all included cases). Only 34.2% (27 cases) of CIN2 biopsies were actual CIN2, 26.6% (21 cases) were CIN1, and 22.8% (18 cases) were non-CIN in their surgical specimens or during follow-up.

Age, colposcopy findings, transformation zone type, and p16 were not associated with the presence of CIN3 or carcinoma in surgical specimens or during follow-up in untreated women. Only Ki-67 and referral cytology were significantly associated with CIN 3 (OR 4.85; 95% CI 1.0–21.97, P 0.02 and OR 0.3, 95% CI 0.06–0.86, P 0.04 respectively). CIN2 biopsies positive for Ki-67 exhibited 4.85 odds of having CIN3 in their surgical specimens or during one year follow-up, whereas HSIL or ASC-H referral cytology exhibited 0.3 odds. (Table 1)

Most of the cases were classified as positive for p16INK4a (81.0%) and negative for Ki-67 (87.2%).

Table 1 shows the utility of p16INK4a and Ki-67 in predicting the presence of CIN3 lesions. Only 17.19% of CIN2 p16INK4a-positive biopsies had CIN3 compared to 40% of Ki-67-positive biopsies. Ki-67 and p16INK4a were negative in 88.24% and 86.67% of non-CIN3 lesions, respectively.

Data on the diagnostic performance of p16INK4a and Ki-67 in identifying the presence of ≥ CIN3 lesions are presented in Table 2. The results showed that p16 staining had high sensitivity and low specificity, whereas Ki-67 staining had low sensitivity and high specificity for detecting coexisting ≥ CIN3. Both markers showed high negative predictive values.

**Table 2.** Diagnostic performance of immunohistochemical expression of p16INK4a and Ki-67 in cervical intraepithelial neoplasia grade 2 biopsies to identify the presence of cervical intraepithelial neoplasia grade 3 in surgical specimens and one-year follow-up

Test	p16INK4a	Ki-67
**Sensitivity**	84.62% (57.76–95.67)	33.33% (13.81–60.94)
**Specificity**	19.70% (11.89–30.84)	90.91% (81.55–95.77)
**PPV**	17.19% (9.87–28.21)	40.00% (16.82–68.73)
**NPV**	86.67% (62.12–96.26)	88.24% (78.46–93.92)
**LR** ^**+**^	1.05 (0.98–1.12)	3.66 (0.99–13.54)
**LR** ^**–**^	0.78 (0.15–3.84)	0.73 (0.57–0.94)

PPV = positive predictive value; NPV = negative predictive value; LR = likelihood ratio.

If LAST recommendations were applied to orient the decision for immediate treatment based only on p16INK4a status, 17 of 23 patients with clinical follow-up would have been treated, but none would have had CIN3. In the treated group, 13 had ≥ CIN3 lesions in the surgical specimens. If the LAST classification had been used in this group, nine patients would not have been treated, of which only two patients had CIN3 (Table 3).

**Table 3:** p16INK4a staining status according to clinical management and final diagnosis

Clinical management	P16INK4A status	Final diagnosis n (%)		Total
		≤ **CIN2** ^a^	≥ **CIN3** ^b^	
**Follow-up**	**Negative**	6 (9.1%)	0	23
	**Positive**	17 (25.8%)	0	
**Treatment**	**Negative**	7 (10.6%)	2 (15.4%)	56
	**Positive**	36 (54.5%)	11 (84.6%)	
**Total**		**66 (100%)**	**13 (100%)**	**79**

^a^ ≤ cervical intraepithelial neoplasia grade 2 (CIN2) includes cervical intraepithelial neoplasia grades 1 or 2 or absence of cervical intraepithelial neoplasia in surgical specimens or during follow up of untreated women; ^b^≥ cervical intraepithelial neoplasia grade 3 (CIN3) includes CIN 3.

If the 50% cut-off of Ki-67 had been applied to orient the clinical decision, only one patient with ≤ CIN2 lesions in the follow-up group would have been treated unnecessarily. In the group treated after CIN2 diagnosis, 46 patients would not have been treated based on the Ki-67 50% cutoff, including eight patients with CIN3 (Table 4).

**Table 4.** Ki-67 staining status according to clinical management and final diagnosis

Clinical management	Ki-67 status	Final diagnosis n (%)		Total
		≤ **CIN2** ^a^	≥ **CIN3** ^b^	
**Follow-up**	**Negative**	22 (33.3%)	0	23
	**Positive**	1 (1.5%)	0	
**Treatment**	**Negative**	38 (57.6%)	8 (66.7%)	55
	**Positive**	5 (7.6%)	4 (33.3%)	
**Total**		**66 (100%)**	**12 (100%)**	**78**

^a^ ≤ cervical intraepithelial neoplasia grade 2 (CIN2) includes cervical intraepithelial neoplasia grades 1 or 2 or absence of cervical intraepithelial neoplasia in surgical specimens or during follow up of untreated women; ^b^≥ cervical intraepithelial neoplasia grade 3 (CIN3) includes CIN 3.

## DISCUSSION

This study assessed the utility of p16INK4a and Ki-67 IHC expression in predicting CIN3 in patients with CIN2 biopsy in their surgical specimens or during follow-up. Positive expression of p16INK4a was defined according to LAST recommendations,^16^ and that of Ki-67 was determined based on a 50% cutoff.^20,21^ The period from 2006 to 2016 was selected because there were no IHC markers available for routine diagnosis, and clinical decisions were made without p16INK4a or Ki-67 staining in our institution.

The high proportion (70.9%) of excisional treatment in our sample reflects adherence to the Brazilian Guidelines for Cervical Cancer Screening, i.e., follow-up in patients younger than 25 years and excisional treatment for CIN2 lesions in patients older than 25 years.^2^ The mean age of patients who underwent excision (34.9 years) and follow-up (26.9 years) were consistent with these guidelines.

The above-mentioned recommendations resulted in 49.4% overtreatment of women with CIN2 biopsy to avoid 16.8% underdiagnosis of CIN3 in our sample; thus revealing the need for other factors to orient treatment decisions in these patients.

The predominance of p16INK4a-positive samples was expected, consistent with a previous study that reported an 84% positive rate for this biomarker.^23^ This high p16INK4a rate is because most CIN2 lesions are associated with high-risk HPV.^24^ This leads to the accumulation of p16INK4a in cells.^25,26^ The predominance of Ki-67-negative cases suggests that the 50% cutoff value used in this study was too high, although it contributed to the high negative prediction of higher-grade lesions.

According to our results, using p16INK4a and Ki-67 is not accurate for deciding between follow-up and immediate treatment in patients with CIN2 biopsies, because most positive cases do not represent CIN3, as observed in another study.^27^

In our study, p16INK4a had a high negative predictive value for CIN2 biopsy (Table 2), which may be useful in avoiding CIN2 overtreatment in women with p16INK4a-negative biopsies. In such cases, the lesion likely involves CIN1 or CIN mimickers. These findings corroborate the suggestion of downgrading histological CIN2 to LSIL when p16INK4a is negative, thereby avoiding overtreatment.^16,28^

Although negative p16INK4a immunostaining ruled out CIN3 or carcinoma in 86.67% of patients with CIN2 biopsies in our study, positive p16INK4a staining did not guarantee that lesions were ≥ CIN3. This was because of the high proportion of p16INK4a-positive samples with ≤ CIN2 lesions in surgical specimens or during follow-up, particularly in untreated patients. Based on our data, negative p16INK4a staining can be useful for preventing overtreatment of patients with CIN2 biopsy; however, positive p16INK4a staining is insufficient for recommending excisional treatment.

If the LAST recommendations were applied to our samples, the number of treatments would have increased (Table 3), with overtreatment of patients who were monitored but did not present with high-grade lesions during follow-up. Similar findings were observed by Thrall et al.,^29^ who reported that patients diagnosed with high-grade squamous intraepithelial lesions (HSIL) increased after following the LAST recommendations in routine diagnostic workup. This increase was more significant in women aged 15–24 years. These findings reinforce our hypothesis that p16INK4a staining should not be the sole factor for determining whether to treat patients diagnosed as CIN2 on biopsy.

Ki-67 is also useful for avoiding overtreatment. Few studies have assessed the potential of Ki-67 for detecting ≥ CIN3 cervical lesions in patients with CIN2 biopsies.^20,21^ Unlike p16INK4a, which was predominantly positive in our study, Ki-67 positivity lead to lower risk of overtreatment of positive cases, as would have been the case with p16INK4a; however, negative Ki-67 might result in increased underdiagnosis of CIN3 (66.7%) [Table 4].

No carcinomas were detected in the current study, although other studies have reported a low risk of cancer (0.2%,^8^ 0.5%^7^) in patients with CIN2 biopsies. Even a low risk of cancer may be unacceptable for some women.^30^ CIN3, when identified, should be treated, and biopsy guided by colposcopy failed to identify concurrent CIN3 in 16.8% of cases. Subsequent diagnosis of CIN3 after CIN2 biopsies in our sample did not represent disease progression, but rather correction of previously misclassified CIN2 or sampling error, including missed CIN3 at colposcopy.^28^ This is a well-known limitation^14–17,30^ that should be considered after a CIN2 biopsy.

Brazilian Guidelines^2^ and other authors define age under 25 or 30 years^3–7^ as the safe limit to avoid treating patients with CIN2 biopsies. In this study, age did not correlate with the presence of CIN3, corroborating previous reports.^8,11^ This unexpected finding can be explained by our inclusion criteria and small sample size. The limitations of our study and that no patients in the untreated group had CIN3 suggest that the criteria used to guide clinical decisions in our setting, as recommended in the Brazilian Guidelines for Cervical Cancer Screening,^2^ appear safe even without these biomarkers.

Another finding that contrasted previous reports,^8,11^ was the 0.3 OR for ASC-H/HSIL compared to negative/LSIL/ASC-US for the presence of CIN3. In our samples, cytological diagnosis was part of screening in primary care units and was not reviewed by pathologists. Another possibility was selection bias because we included samples with CIN2 biopsy, which usually occurs when altered colposcopy does not confirm the cytological diagnosis and a biopsy is needed for a more accurate evaluation. This limitation may also explain insufficient association between other factors and CIN3 in our study.

One possible limitation of this study was using samples from a referral center for cervical pathology. These markers may be more useful in studies involving community pathologists,^15^ since overinterpretation of immature squamous metaplasia as CIN2, which is the most frequently reported cause of negative excisional procedures after a high-grade lesion on biopsy,^31^ may be more common than when specimens are analyzed by specialized pathologists.

Another potential limitation was the number of exclusions and losses to follow-up of untreated patients. Missing data (10 patients) and absence of lesions on IHC slides (4 patients) led to the exclusion of 14 patients from our study; however, since these exclusions were unrelated to the outcome, they were probably not a source of bias. However, excluding 28 cases in which biopsy was defined as excisional should be considered. If these cases had similar p16INK4a-positive rates, their subsequent histology, if treated or followed-up when untreated, would probably have been negative, thereby altering our findings. This strategy was chosen because, in our setting, treating excisional biopsies showing CIN2 is not a cause for treatment, especially in women younger than 25 years, as in some patients with small lesions and squamous columnar junctions completely seen.

The cases in which CIN2 was not confirmed (25 biopsies) illustrate the discrepancy between initial histological diagnosis and reviewed diagnosis in the present study and reinforce the moderate interobserver agreement in CIN2 biopsies. This limitation must be considered when deciding whether to treat these patients.

Considering the very low number of patients lost to follow-up in our patient sample, this did not appear to result in a selection bias.

Our findings represent the experience of a single institution in managing CIN2 biopsied patients and should not be considered alone in orienting treatment decisions for these women. Additionally, we did not assess the risk of disease progression in untreated women. Despite these limitations, our study can be useful in avoiding the misuse and misinterpretation of p16INK4a and Ki-67 in CIN2 biopsies.

Detecting CIN2 lesions reflects the variability in the biological behavior of HPV-induced lesions, in which not all lesions undergo the molecular changes necessary for classification as preneoplastic and represent a transitory HPV infection with spontaneous resolution. CIN2 also has a lower risk of progression to carcinoma compared to CIN3,^3,32^ which leads us to view CIN2 as a lesion with unknown behavior. Therefore, consistent with previous studies,^5,28^ we consider it safer for pathologists to continue using the three-tier diagnostic category of CIN (CIN1, CIN2, CIN3) rather than the two-tier classification (LSIL × HSIL) for biopsy samples.

Strategies to identify patients diagnosed with CIN2 and lower risk of CIN3 are helpful for preventing overtreatment, especially in women of reproductive age, and for limiting the underdiagnosis of higher-grade lesions. Such strategies are required to improve management of CIN2. Until better strategies are available, we agree with Cruickshank that patients with CIN2 biopsies should be fully informed of the risks and benefits of all management options before being treated.^30^ In this approach, we believe that biomarker status should be considered adjuvant information rather than the sole factor for determining whether to treat these patients. However, in cases where the squamous columnar junction cannot be seen entirely, or where the patient has difficulty remaining in active surveillance, treatment should be considered.

## CONCLUSIONS

Negative p16INK4a and Ki67 IHC staining can assure the absence of higher-grade lesions in more than 85% of patients with CIN2 biopsies and should, thus, be used to prevent overtreatment of these patients. Positive p16INK4a and Ki-67 immunohistochemical staining did not predict CIN3 in patients with CIN2 biopsies.
